# Behavioral Phenotyping of Dopamine Transporter Knockout Rats: Compulsive Traits, Motor Stereotypies, and Anhedonia

**DOI:** 10.3389/fpsyt.2018.00043

**Published:** 2018-02-22

**Authors:** Stefano Cinque, Francesca Zoratto, Anna Poleggi, Damiana Leo, Luca Cerniglia, Silvia Cimino, Renata Tambelli, Enrico Alleva, Raul R. Gainetdinov, Giovanni Laviola, Walter Adriani

**Affiliations:** ^1^Center for Behavioral Sciences and Mental Health, Istituto Superiore di Sanità, Rome, Italy; ^2^Department of Neuroscience, Istituto Superiore di Sanità, Rome, Italy; ^3^Neuroscience and Brain Technologies Department, Fondazione Istituto Italiano di Tecnologia, Genoa, Italy; ^4^Faculty of Psychology, International Telematic University Uninettuno, Rome, Italy; ^5^Department of Dynamic and Clinical Psychology, Sapienza University of Rome, Rome, Italy; ^6^Skolkovo Institute of Science and Technology, Moscow, Russia; ^7^Institute of Translational Biomedicine, St. Petersburg State University, St. Petersburg, Russia

**Keywords:** Intolerance-to-Delay Task, sucrose consumption, appetitive food eating, reward sensitivity, behavioral rigidity

## Abstract

Alterations in dopamine neurotransmission are generally associated with diseases such as attention-deficit/hyperactivity disorder (ADHD) and obsessive-compulsive disorder (OCD). Such diseases typically feature poor decision making and lack of control on executive functions and have been studied through the years using many animal models. Dopamine transporter (DAT) knockout (KO) and heterozygous (HET) mice, in particular, have been widely used to study ADHD. Recently, a strain of DAT KO rats has been developed (1). Here, we provide a phenotypic characterization of reward sensitivity and compulsive choice by adult rats born from DAT–HET dams bred with DAT–HET males, in order to further validate DAT KO rats as an animal model for preclinical research. We first tested DAT KO rats’ sensitivity to rewarding stimuli, provided by highly appetitive food or sweet water; then, we tested their choice behavior with an Intolerance-to-Delay Task (IDT). During these tests, DAT KO rats appeared less sensitive to rewarding stimuli than wild-type (WT) and HET rats: they also showed a prominent hyperactive behavior with a rigid choice pattern and a wide number of compulsive stereotypies. Moreover, during the IDT, we tested the effects of amphetamine (AMPH) and RO-5203648, a trace amine-associated receptor 1 (TAAR1) partial agonist. AMPH accentuated impulsive behaviors in WT and HET rats, while it had no effect in DAT KO rats. Finally, we measured the levels of tyrosine hydroxylase, dopamine receptor 2 (D2), serotonin transporter, and TAAR1 mRNA transcripts in samples of ventral striatum, finding no significant differences between WT and KO genotypes. Throughout this study, DAT KO rats showed alterations in decision-making processes and in motivational states, as well as prominent motor and oral stereotypies: more studies are warranted to fully characterize and efficiently use them in preclinical research.

## Introduction

Brain dopamine (DA) is closely involved in the modulation of several neurobiological and behavioral processes, including *decision making*, reward processing, motivational states, habits, and movement control. Dysregulation in forebrain dopaminergic signaling and function is associated with many neurological and neuropsychiatric diseases, such as schizophrenia (SZ), attention-deficit/hyperactivity disorder (ADHD), obsessive-compulsive disorder (OCD), and Parkinson disease (PD) (2–5). Encoded by the SLC6A3 gene, dopamine transporter (DAT) has a key role in reuptake; therefore, its activity is fundamental for a correct regulation of dopaminergic pathways. The importance of DAT activity in DA circuitry is supported by the effects of many drugs of abuse and neurotoxins; current pharmacological therapeutic strategies for diseases like ADHD or a major depression involve drugs such as psychostimulants or triple reuptake inhibitors (6–8).

Psychostimulants such as amphetamine (AMPH) or methylphenidate can, however, have an effect also on trace amine-associated receptor 1 (TAAR1), another protein that has a significant role in DA neurotransmission. TAAR1 is a G protein-coupled receptor that colocalizes with DAT, has close interactions with it, and can modulate its activity when activated (9, 10). At the same time, TAAR1 modulates D2 DA receptor function likely due to heterodimerization (11, 12). Intriguingly, altered levels of trace amines have been associated with many diseases, including ADHD. Moreover, it has been suggested that most of the effects of psychostimulants may be modulated by influencing TAAR1 activity (13, 14). TAAR1 is therefore proposed as a target for new medications, and various compounds are under preclinical development. Research in this field needs to move across preclinical studies in animal models, and this allows direct insights into the neurobiological, genetic, and biopsychological functioning, in the aforementioned mental diseases.

Several of the main features issuing from an impairment of DAT activity in humans have been studied using the DAT knockout (KO) mouse (15–17). Since first generated, DAT KO mice have been used in a wide range of studies, thanks to their peculiar behavioral phenotype. Specifically, in addition to early life mortality, the DAT KO mice show hyperactivity, cognitive deficits, sleep dysregulation, and a low behavioral inhibition. They also have relevant alterations in dopaminergic tone within frontostriatal circuits and in the striatal expression of many proteins, such as D1 and D2 receptors and tyrosine hydroxylase (TH). Finally, they also show a paradoxical reduction of locomotor activity after AMPH treatment [see (17–19)]. Because of such features, DAT KO mice have been extensively used as a model of ADHD, a widespread neurodevelopmental disorder affecting up to 2–5% of infants and adolescents and persisting into adulthood (20, 21).

Well known to be comorbid with other diseases such as major depression and bipolar disorder (22), ADHD has reportedly been associated with many other psychiatric disorders such as behavioral addictions, OCD, oppositional defiant disorder, and conduct disorder (CD) (23), all of which share common symptoms such as poor decision making and impaired impulse control. DAT KO mice, however, have proven useful also in understanding many other neuropsychological diseases, like SZ (24, 25) or bipolar disorder (26). Recently, DAT KO mice have been used to model human DAT deficiency syndrome (DTDS), also known as early PD, a rare Parkinsonian-like movement disorder. DTDS’ symptoms can range from dystonic muscle contractions to bradykinesia, postural instability, and hypomimia: these are believed to be caused by an impaired DAT functionality, due to mutations in the SLC6A3 gene (27–30). It should be noted that due to the extreme phenotype of DAT KO mice, which could influence or even impair some behavioral measures, they have been avoided in some recent studies in favor of heterozygous (HET) DAT hypofunctional mice. These mice show milder but clear behavioral alterations, especially during adolescence: such phenotype consists of a motor hyperactivity, attentional deficits, impulsive behaviors, and an AMPH-mediated rescue, consistent with the construct of ADHD (31, 32).

Recently, Gainetdinov et al. developed a novel DAT KO rat model that could provide a good translational model for ADHD as well as other human diseases involving alterations in DA circuitry. Their first work on DAT KO rats (1) showed increased extracellular DA concentrations, hyperactivity and cognitive deficits, as well as dysregulation in frontostriatal BDNF function. More in detail, DAT KO rats develop normally but weigh less than HET and wild-type (WT) rats, have a pronounced spontaneous hyperactivity, and demonstrate a deficit in working memory and in sensori-motor gating. While striatal extracellular DA concentrations are significantly increased, the total tissue content of DA is markedly decreased (1). The hyperactivity of DAT KO rats can be counteracted by AMPH, methylphenidate, and a few other compounds, as well as by transient DAergic rearrangement typical of adolescent age (33). Here, we provide evidence of other behavioral alterations affecting reward processing, decision making, and impulse control in DAT KO rats. First, we tested whether DAT KO rats have an abnormal sensitivity to rewarding stimuli, and then we tested their cognitive impulsivity through the use of the Intolerance-to-Delay Task (IDT). Finally, we measured the basal gene expression in ventral striatum to check whether DAT KO rats have a different expression of genes associated with monoaminergic signaling.

The IDT is an operant task that involves a choice between either an immediate small reward or a delayed larger reward (in the form of smaller or larger quantities of food), extensively used to study behavioral impulsivity in rodents (34). The two options are calibrated so that the first one gives an immediate benefit, but is suboptimal in terms of payoff (i.e., global food gain), while the latter gives an overall optimal benefit throughout the whole task (35, 36). Through the process of temporal discounting, increasing the delay associated with the larger reward results in a progressive shift of preference from the larger to the smaller reward; subjects with a higher cognitive impulsivity tend to shift their preference even with short delays while subjects persisting on large-reward choice despite suboptimal payoff may be labeled as compulsive (35–38). We also studied the effects of AMPH and of a partial TAAR1 agonist drug during the execution of the IDT as a function of DAT genotype.

## Materials and Methods

All experimental procedures were approved by the Italian Ministry of Health (formal license 5/2014-B, to GL). Procedures were in close agreement with the European Community Council Directive (2010/63/EEC) and Italian law. All efforts were made to minimize animal suffering, to reduce the number of animals used, and to utilize alternatives to *in vivo* techniques, if available.

### Subjects

A total of 39 adult (8-month-old) male rats (with a Wistar Han background), of which 10 were WT (average body weight 530 g), 17 HET (average body weight 530 g), and 12 DAT KO (average body weight 305 g), were used. These animals were obtained from Istituto Italiano di Tecnologia (IIT) (Genova, Italy), with the purpose of setting a colony at Istituto Superiore di Sanità (ISS) (Rome, Italy). Animals were born in IIT from the breeding of DAT–HET males and females, weaned on PND 21 and shipped to ISS at adult age, where they were housed in pairs within Makrolon^®^ III cages with sawdust bedding and with food and water *ad libitum*. No more than a pair of sibling subjects per genotype was chosen to be shipped, in order to avoid possible genetic biases. All dams being DAT–HET and all three genotypes being represented in each litter, there is no risk of biases due to differential maternal behavior. They were kept in an air-conditioned room (temperature 21 ± 1°C, relative humidity 60 ± 10%), on a 12-h reversed light–dark cycle (lights off at 07:00 a.m.) until the start of the experimental protocols.

During the first week, a cohort of 24 subjects (8 WT, 8 HET, and 8 KO) was randomly selected, and they underwent a test to measure their preference for a sweet solution compared to water and a test to study their behavior when exposed to a highly appetitive food. Rats were housed individually within Makrolon^®^ III cages with sawdust bedding for the duration of these procedures only. Afterward, all 39 rats were tested in an IDT, for a total of 6 weeks of testing. During this experimental protocol, a food restriction, to keep subjects at 95% (±2%) of their body weight, was imposed through a limited quantity of extra food given at the end of each IDT free-feeding experimental session. This strategy was applied to increase the animals’ motivation to work for food delivery at the beginning of each session.

### Body Weight and Food Consumption

Body weight was measured thrice in a 2-week span before the start of the experimental protocols. Food consumption was measured during the same 2 weeks, by weighing the food pellet present in each home cage tray every 2 days. During the IDT, subjects’ body weight was measured three times per week, for the whole duration of the experimental protocol, in order to monitor and to titrate the level of food restriction.

### Exp. 1: Preference for a Sweet Solution

During 4 days of testing, each rat had free access to two different bottles in its home cage, one containing water and one containing a sucrose solution (14%). The starting position of each bottle was counterbalanced, and each day the position of the bottles was swapped. The amount of fluid intake was measured for each subject by weighing each bottle every day at the same hour, with a Kern 440-49 N balance (Kern & Sohn, Balingen, Germany).

### Exp. 2: Exposure to a Highly Appetitive Food

During 4 days of testing, twice per day (10:30 a.m. and 12:00 p.m.), each rat received 4.5 g of mascarpone cheese in its home cage, delivered inside baking paper cups and left on the cage floor for 10 min. After this time, both baking cups and remaining cheese were removed with the quantity of remaining cheese and the condition of the baking cups being measured. The remaining cheese was quantified as “all,” “more than half,” “less than half,” “none.” The condition of the cups was classified as “present” if it was left intact, and “deteriorated” or “absent” if it was destroyed or eaten.

### Exp. 3: Intolerance-to-Delay Task

#### Apparatus

Eight operant panels (HOPs, PRS Italia, Rome, Italy) were each placed in a Makrolon^®^ III cage with sawdust bedding: therefore, the apparatus consisted of standard cages identical to the subjects’ home cages. The panels, which occupied one quarter of the living area, were provided with two nose-poking holes; two hole lights, placed inside each nose-poking hole; one feeding magazine where precision food pellets (45 mg, F0021, BioServ, Frenchtown, NJ, USA) were dropped, placed centrally between the nose-poking holes; one magazine light; one main chamber light, placed at the top of the panel; one hidden-feeding device that released food pellets into the magazine. Operant panels were connected through an interface to a computer. The whole task procedure was monitored and recorded through software (Sk020, PRS Italia, Rome, Italy).

#### Procedure

Subjects were tested for 5 days per week between 10:00 a.m. and 14:00 p.m., for a total of 15 training and 15 test sessions performed during a 6-week span. Each rat was tested in the same chamber at the same hour, and sessions lasted for 40 min. During each session, subjects could nose-poke into either of the two holes to release pellets in the magazine. Nose-poking in one hole [termed small and soon (SS)] resulted in the immediate delivery of one pellet in the magazine, whereas nose-poking in the other hole [termed large and late (LL)] resulted in the delivery of five pellets in the magazine after a set delay, whose length was 0 s during training, 15 s during test week 1, 30 s during test week 2, and 45 s during test week 3. Lights were used as visual cues: the main light was on to signal whenever the panel could be activated by a nose-poke; the hole lights were switched on just after a nose-poke in the corresponding hole for 1 s until food delivery. After any food delivery, the panel entered a timeout (TO) of 30 s, during which additional nose-poking was recorded but was without any consequence (inadequate nose-pokes); the magazine light was kept switched on for the entire duration of the TO.

#### Training Phases (First 3 Weeks of IDT)

During a first “2vs2” training phase (on the first week of IDT testing), nose-poking in either hole resulted in the immediate release of two pellets. This phase was used to let subjects familiarize with the apparatus with no reason to prefer any of the two holes. During the second “1vs5” training phase (on the second and third weeks of IDT testing), SS nose-pokes resulted in the release of one pellet, whereas LL nose-pokes resulted in the release of five pellets (but with a delay of 0 s). This phase allowed subjects to reach a clear preference for LL. During this phase, two animals did not reach a preference for LL; thus, they were excluded from data analysis.

#### Drugs

We used d-AMPH (1 mg/kg i.p. dissolved in saline solution) and RO-5203648 (39), a partial agonist of TAAR1 receptors, obtained from F. Hoffmann la Roche Ltd., Basel, Switzerland (1 mg/kg i.p. dissolved in a vehicle, i.e., DMSO 1% in distilled water). During 1vs5 training phase, every subject received saline i.p. before each session. During the three testing weeks, on the first day of every test week (Monday), every subject received saline i.p. before the session. The following 4 days of every test week (Tuesday–Friday), before each session, every subject received one of the following: saline, DMSO 1%, AMPH, or RO-5203648.

Amphetamine or saline was injected 15 min before session start, and RO-5203648 or vehicle was injected 10 min before session start. Therefore, both drugs had their peak effect during the middle of sessions. AMPH and RO-5203648 were administered on random days, but never administered on subsequent days; this produced eight different combinations of administration order during a test week, which was randomly assigned to subjects. Each combination was used on average on five subjects (at least one for each genotype), counterbalancing between genotypes to minimize any bias related to a specific administration order.

### Genotyping by RFLP

The WT, DAT–HET, and DAT KO genotypes were checked on purified DNA by restriction fragment length polymorphisms (RFLPs): indeed, while the WT allele has one BtsIMutI restriction site, this was specifically canceled in the KO allele (1). The DNA of each sample was extracted from a piece of tail of each rat, using the Dneasy Blood & Tissue Kit (QIAGEN) according to the manufacturer’s protocol. The target region was amplified using the following primer sequences: forward 5'-TCC TGG TCA AGG AGC AGA AC-3' and reverse 5'-CAC AGG TAG GGA AAC CTC CA-3'. These primers and the enzyme BtsIMutI (New England BioLabs, Ipswich, MA, USA) were used to identify the genotypes.

PCR amplification was performed in a 50-µl reaction volume. After the initial denaturation at 94°C for 15 min, there were 35 cycles at 95°C for 30 s, 58°C for 30 s, and 72°C for 30 s, and then a final extension step of 72°C for 5 min. The DNA was substituted with sterile deionized water for the negative control. The PCR products were resolved on 2% agarose gel and identified by Novel Juice (GeneDirex) staining. For the genotypes analysis, 10 µl of the PCR products was digested for 1 h at 55°C with 1 µl of BtsIMutI (10 U/μl) in separate tubes. The cleaved products were resolved on 2.5% agarose gel.

### RT-PCR and *Ex Vivo* Markers

Two weeks after the last behavioral test, 10 WT and 12 KO rats were sacrificed in a random order through decapitation in two consecutive days between 10:00 a.m. and 12:00 p.m. Brains were quickly removed and dissected on ice with single-use metal scalpels. A 1-mm thick slice of ventral striatum was collected from each brain, with slices immediately stored in 1.5-ml Eppendorf tubes containing 500 µl of RNA stabilization reagent RNAlater (QIAGEN). Samples were taken specifically from the ventral striatum as extensive neurochemical analyses already confirmed the expected changes within the dorsal striatum (1).

Samples were stored at −80°C until processed. Samples were processed to extract the total RNA using Rneasy Mini Kit (QIAGEN) according to manufacturer’s recommended protocol. Briefly, samples were solubilized and homogenized in RLT lysis buffer, loaded in columns containing silica membranes and subjected to washing and centrifugation processes. RNA was eluted in 100-µl volume and snap-frozen at −80°C. The concentration and purity of RNA samples were determined using a NANODROP 2000 (Thermo Scientific).

The cDNA for each sample was obtained using RT^2^ First Strand Kit (QIAGEN) following producer’s recommended protocol. The RNA quantity for each sample was adjusted to 5 ng/µl. Afterward, any genomic contamination was removed by mixing 8 µl from each sample (total of 40-ng RNA) with 2 µl of genomic DNA elimination buffer (QIAGEN) and incubating at 42°C for 5 min. Finally, the retrotranscription was performed on each sample: 10 µl of each sample was mixed with 10 µl of Oligo(dT) retrotranscription mix and incubated at 42°C for 15 min and at 95° for 5 min in a T100 (BIO-RAD) thermocycler. In the same way, negative retrotranscription controls (without retrotranscriptase enzyme) were performed. cDNA samples were then loaded in a total of six custom 96-well RT-PCR plates (QIAGEN). Each of the first six rows of each plate was used for a different assay: TH, TAAR1, Slc6a4 serotonin transporter (SERT), DRD2 (D2), plus the two housekeeping genes 18SrRNA and β-actin. The last two rows were used as positive and negative controls. Each sample was assayed in triplicate; thus, a total of four samples were assayed on each plate. Each well was loaded with 25 µl of solution containing 11.5 µl of water, 12.5 µl of SYBR Green (QIAGEN) mastermix, and 1 µl of cDNA. Amplification was carried out in an ABI 7500 (Applied Biosystem) thermocycler with the following conditions: 95°C for 15 min, followed by 40 cycles (95°C for 15 s and 60°C for 1 min), after the amplification, a melting curve was obtained.

### Behavioral and Molecular Data Analysis

Data were analyzed using repeated measures analysis of variance. For food consumption, we used a one-way model, where the dependent variable was the total food intake and the factor was three-level genotype (WT vs HET vs KO). For preference for a sweet solution, we used a one-way model where the dependent variable was the percentage of a sweet solution intake over the total fluid intake and the factor was three-level genotype (WT vs HET vs KO). For the exposure to a highly appetitive food, we used a logistic multinomial regression for the quantity of cheese and a logistic regression for the cup condition. For IDT, we used a split-plot model, and the factors were three-level genotype (WT vs HET vs KO) × four-level treatment (Sal vs DMSO vs Amph vs RO-5203648) × three-level delay (15 vs 30 vs 45 s). Statistical analysis was performed using StatView II (Abacus Concepts, CA, USA). Data are expressed as mean ± SEM. Significance level was set at *P* ≤ 0.05, NS = not significant. Multiple *post hoc* comparisons were performed with Tukey’s HSD test.

RT-PCR curves were analyzed using ABI 7500 software v2.0.6. Quantification was carried out measuring the number of amplification cycles needed to cross a 0.04 fluorescence threshold (Ct). Melting curve analysis was then carried out to validate the purity of amplification products, and any replica with multiple or abnormal melting temperature was excluded from analysis. For TH, SERT, and D2 genes the cutoff was fixed at 37 cycles. Because of the extremely weak expression of TAAR1, the cutoff was fixed at 39 cycles for such gene. Data for TH, TAAR1, Slc6a4, and Drd2 genes were normalized to the mean of 18SrRNA and β-actin genes for each sample. The relative expression of each gene transcript was obtained using ΔΔCt and converted in the relative expression ratio (2^−ΔΔCt^). The statistical analysis was performed using Microsoft Excel on normalized expression levels of WT vs KO samples. Significance was assessed using a two-tails Student’s *t*-test. Significance level was set at *P* ≤ 0.05, NS = not significant.

## Results

### Body Weight and Food Consumption

**Figure 1:**
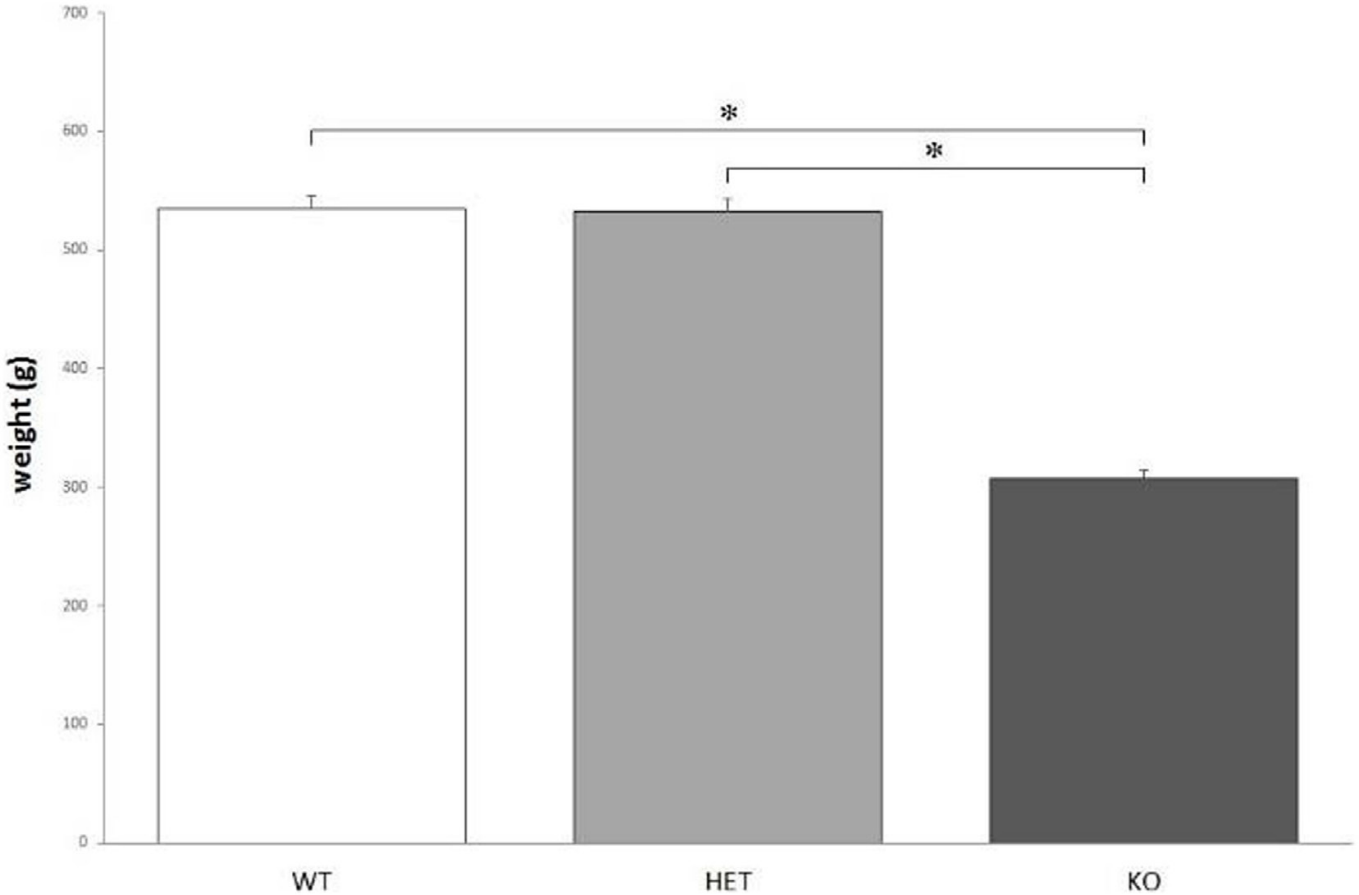
The average body weight measured for the three genotypes. Knockout (KO) rats weigh significantly less when compared to both wild-type (WT) and dopamine transporter–heterozygous (HET) rats. *P < 0.0001.

According to the measures, the weight of KO rats (308 ± 5 g) is significantly lower, when compared to both WT (535 ± 9 g) and DAT–HET (532 ± 6 g) rats: *F*[2, 36] = 130.178, *P* < 0.0001 (Figure 1). The weight difference was confirmed during the whole set of procedures.

Nevertheless, there were no significant differences between genotypes when considering *ad libitum* food intake: *F*[2, 21] = 0.283, *P* = NS.

### Preference for a Sweet Solution

**Figure 2:**
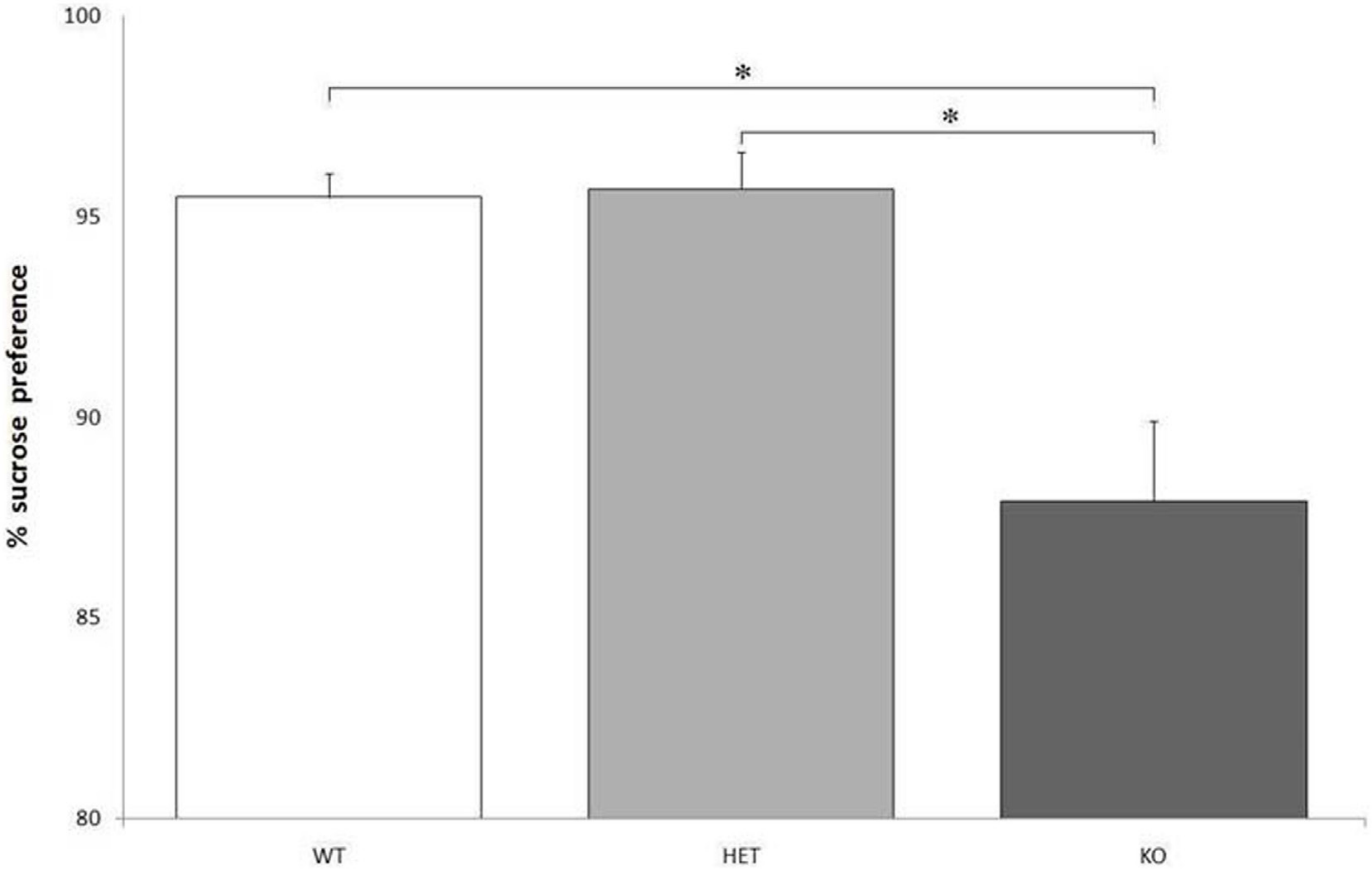
Proportion of the fluid intake from a bottle containing 14% sucrose over tap water. Preference for the sucrose bottle is significantly lower for knockout (KO) rats when compared to both wild-type (WT) and dopamine transporter–heterozygous (HET) rats. *P < 0.0001.

Though there were no significant differences between genotypes when considering the total fluid intake, *F*[2, 21] = 0.129, *P* = NS, KO rats showed a markedly lower preference for the bottle containing a sweet solution, compared to WT and DAT–HET: *F*[2, 21] = 24.216, *P* < 0.0001 (Figure 2).

### Exposure to a Highly Appetitive Food

Multinomial logistic regression analysis showed that KO rats have a higher chance to eat less cheese when compared to both WT and DAT–HET. Considering the four categories for the quantification of the remaining cheese after 10 min (“all,” “more than half,” “less than half,” “none”), KO had a higher chance to leave “all” the cheese compared to leave “none” (*P* < 0.05) and a higher chance to leave “more than a half” compared to leave “none” (*P* < 0.05), when compared to both WT and DAT–HET. Logistic regression analysis showed that KO rats have a higher chance to destroy the paper cups containing the cheese, when compared to both WT and DAT–HET. Considering the categories for the state of paper cups after 10 min (“present” or “deteriorated”/“absent”), KO had a higher chance to destroy the cups (*P* < 0.05), when compared to both WT and DAT–HET.

### Intolerance-to-Delay Task

Training: two KO rats were excluded from statistical analysis, as they did not reach a preference for LL; instead, they showed a high and stable preference for SS throughout the whole training phase. There was an overall significant difference in total nose-pokes between KO (211 ± 62) and the other two groups (WT: 154 ± 8; DAT–HET: 154 ± 11): *F*[2, 12] = 2.055, *P* < 0.05.

#### Latency

Knockout rats showed a higher latency to approach SS in each session, when compared to WT and DAT–HET: *F*[2, 34] = 12.351, *P* < 0.0001. This difference was not present when considering latency to approach LL. There was a significant effect of delay on latency: for all three genotypes, at higher delays, there was a trend toward reduction of the latency to approach SS: *F*[2, 68] = 2.834, 0.05 < *P* < 0.1 and a significant increase of the latency to approach LL: *F*[2, 68] = 5.783, *P* < 0.05. Treatment with both AMPH and RO-5203648 caused a trend toward reduction to approach LL: *F*[3, 102] = 2.577, 0.05 < *P* < 0.1.

#### LL Preference

**Figure 3:**
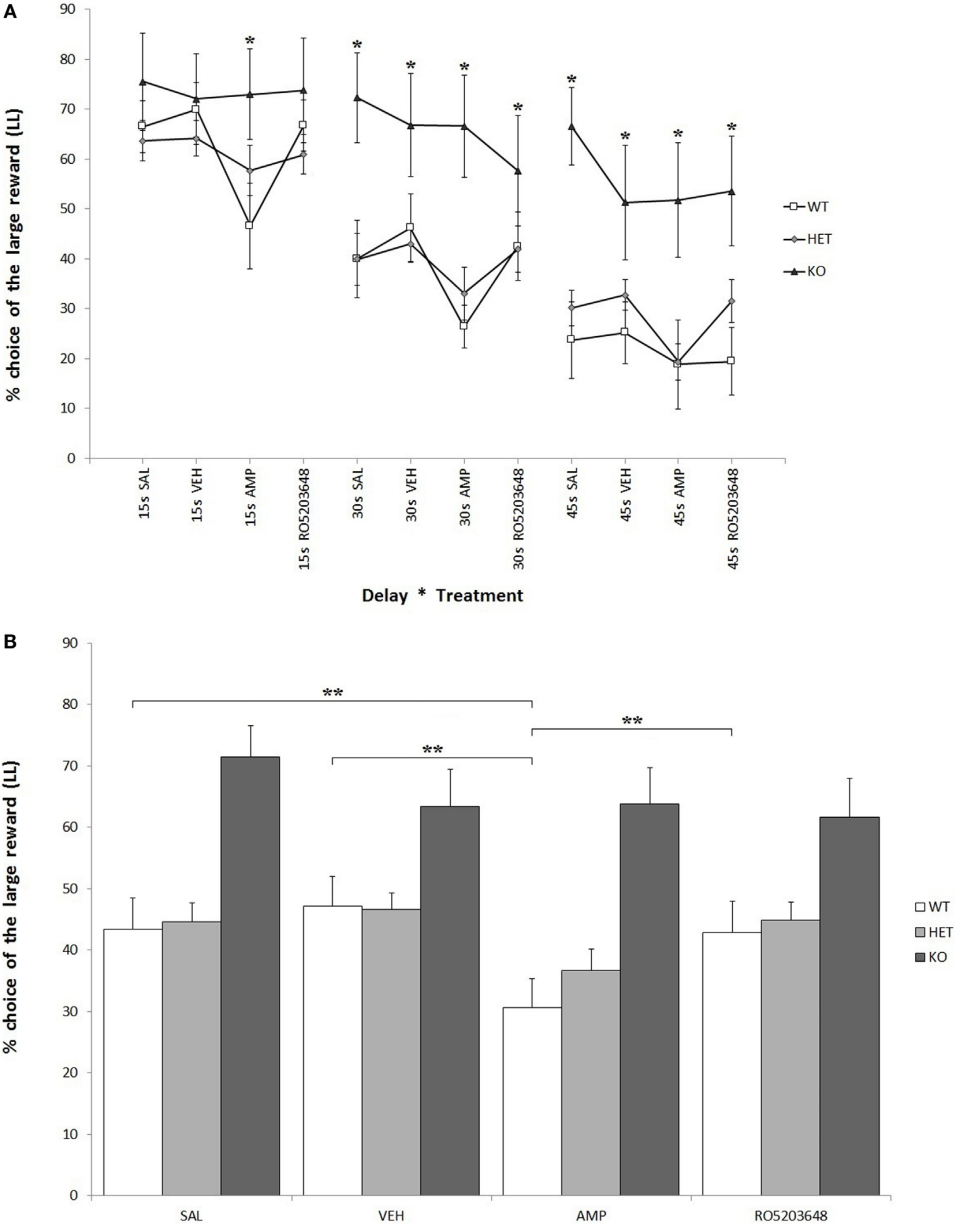
Percentage preference for a large-and-late (LL) reward over a small-and-soon (SS) one in an operant Intolerance-to-Delay Task. **(A)** Preference for LL at increasing delays after the administration of different drugs. Whereas preference for LL decreases significantly at higher delays, knockout (KO) rats show a significantly higher preference for LL when compared to both wild-type (WT) and dopamine transporter–heterozygous (HET) rats. Amphetamine (AMPH) reduces significantly LL preference in WT rats at all three delays. *P < 0.05. **(B)** The effect of the different drugs on LL preference (average value collapsed across delays). WT rats, after AMPH administration, show a significantly reduced LL preference compared to the saline, vehicle, and RO-5203648 groups. **P < 0.0001.

As expected, there was a strong effect of delay on LL preference for all three genotypes. At higher delays, there was a significant reduction in LL preference: *F*[2, 68] = 44.597, *P* < 0.0001. This reduction was, however, less pronounced when considering only KO rats: *F*[2, 4] = 2.133, 0.05 < *P* < 0.1. In other terms, KO rats showed a higher preference for LL throughout the whole testing phase: *F*[2, 34] = 6.644, *P* < 0.05 (Figure 3A). There was a significant effect of treatment, especially AMPH, on reducing LL preference: *F*[3, 102] = 8.611, *P* < 0.0001. This effect was stronger in WT, slightly less pronounced in DAT–HET, and absent in KO rats: *F*[3, 6] = 2.448, *P* < 0.05 (Figure 3B). This drug effect was evident at all delays; however, although there was no significant treatment by delay interaction, *post hoc* analyses revealed differential extent of AMPH effects: in WT rats, AMPH-decreased LL preference emerged at 15 and 30 s, but not at 45 s; in DAT–HET rats, AMPH-decreased LL preference emerged at 45 and 30 s, but not at 15 s (Tukey’s HSD, Ps < 0.05). Therefore, WT rats seem to show some tolerance to AMPH effects at a 45-s delay, which can well be interpreted as a floor effect, whereas DAT–HET rats seemed conversely to display sensitization at a 45-s delay, which can well be interpreted as a subchronic effect.

#### Number of Trials

**Figure 4:**
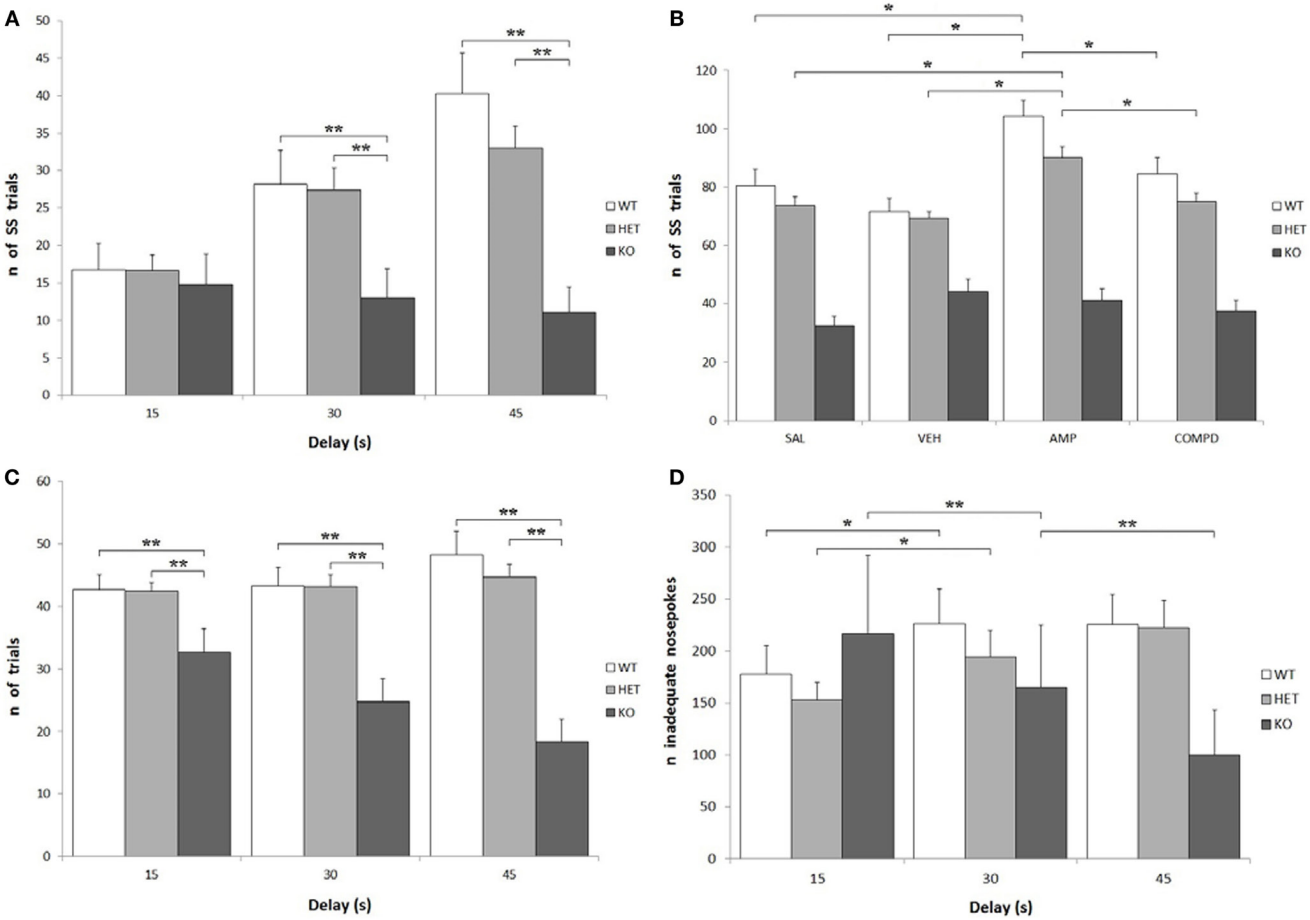
Other parameters from the Intolerance-to-Delay Task. **(A)** Total small-and-soon (SS) trials at increasing delays. While there is a clear increase of the number of SS trials at higher delays in wild-type (WT) and dopamine transporter (DAT)–heterozygous (HET) rats, knockout (KO) rats did not show such an increase. **P < 0.0001. **(B)** The effect of the different drugs on the total SS trials. The administration of amphetamine (AMPH) on WT and DAT–HET rats has a significant effect to increase the total SS trials, if compared to saline, vehicle, and RO-5203648. *P < 0.05. **(C)** Total [SS + large-and-late (LL)] trials performed at all the three delays: total trials of KO rats were always significantly lower than those of WT and DAT–HET rats. **P < 0.0001. **(D)** The total inadequate nose-pokes, performed in both nose-poking holes during the timeout (i.e., when without consequences). For WT and DAT–HET rats, the total inadequate nose-pokes significantly increase at higher delays; conversely, for KO rats, inadequate nose-pokes significantly decrease at higher delays. *P < 0.05, **P < 0.0001.

Knockout rats showed a significantly lower number of total effective nose-pokes (i.e., in both holes) when compared to both WT and DAT–HET: *F*[2, 34] = 27.599, *P* < 0.0001. There was also a significant effect of delay on the number of total trials: while KO rats showed a significant decrease of trials at higher delays, WT rats conversely showed an increase of trials: *F*[4, 68] = 12.758, *P* < 0.0001 (Figure 4C). Treatment, especially AMPH, caused a reduction of total trials at low delays (15 s), while it caused an increase of total trials at higher delays (30 and 45 s): *F*[6, 204] = 6.105, *P* < 0.0001 independently from genotype. KO rats performed fewer SS trials than both WT and DAT–HET: *F*[2, 34] = 12.947, *P* < 0.0001. This is consistent with the overall enhanced LL preference in this genotype. There was also a significant genotype by delay interaction: *F*[4, 68] = 9.878, *P* < 0.0001, as such scarcity became even more pronounced at higher delays (Figure 4A). Finally, there was also a significant genotype by treatment interaction: *F*[6, 102] = 4.032, *P* < 0.05. Specifically, AMPH caused an increase of SS nose-pokes in WT and DAT–HET rats (Figure 4B). Considering only LL trials, there was a significant effect of delay: as expected, higher delays caused a reduction in LL trials for all three genotypes: *F*[2, 68] = 95.766, *P* < 0.0001.

#### Number of Inadequate Nose-Pokes

As expected, there was a significant increase in the total inadequate nose-pokes in both WT and DAT–HET at higher delays, while KO showed an opposite trend, with a significant decrease of inadequate nose-pokes: *F*[4, 68] = 10.096, *P* < 0.0001 (Figure 4D). This generalized decrease in KO nose-poking (both inadequate and adequate, see above) was correlated with the appearance of noticeable motor stereotypies (sniffing, chewing) aimed at the edges of the magazine (unformal observations during a task). This profile seems to suggest that KO rats reacted to delay increase with repetitive/compulsive behaviors, rather than motoric/impulsive ones. There was a general increase in SS inadequate nose-pokes at higher delays: *F*[2, 68] = 14.856, *P* < 0.0001; however, such an increase interacted with genotype, as it was observed only in WT and DAT–HET and not in KO: *F*[4, 68] = 8.045, *P* < 0.0001. In other words, KO rats did not display restlessness at high delays. Treatment, especially AMPH, caused a reduction in SS inadequate nose-pokes at lower delays (15 s), while it caused an increase of SS inadequate nose-pokes at higher delays (30 and 45 s): *F*[6, 204] = 3.972, *P* < 0.05. No significant genotype by treatment interaction was shown for SS inadequate: *F*[6, 102] = 1.495, *P* = NS. There was a significant decrease in LL inadequate nose-pokes at higher delays: *F*[2, 68] = 14.914, *P* < 0.0001, and this decrease was much more pronounced in KO rats when compared to both WT and DAT–HET: *F*[4, 68] = 4.203, *P* < 0.05. In other words, KO rats reacted to a delay by showing a choice for LL in the fewer trials with no persistence onto LL hole during TO. Treatment, especially AMPH, caused a significant decrease in LL inadequate nose-pokes, for all the three genotypes: *F*[3, 102] = 10.601, *P* < 0.0001.

### Confirmation of Genotype

First of all, we underline that an extensive proof about the effectiveness and consequences of DAT-abolished allele(s) can be found elsewhere (1). Just by informal observation in the home cages, DAT KO rats demonstrate evident hyperactivity, with compulsive rearing at the four corners of the cage and stereotyped sniffing directed against focal items of the cage grid. Also, while other animals are found asleep or resting after 1 h following the switch on of facility lights, the DAT KO rats were always found to be still awake and running around at that time.

In our RFLP analysis, the WT genotype undergoes cutting on both alleles and produces two low digested bands (104 and 71 bp); in the DAT–HET genotype, only one of the alleles is cut, resulting in one undigested and two digested bands (175, 104, and 71 bp); the KO genotype loses all cutting sites on both alleles and displays only one undigested band (175 bp). The genotypes of all rats used in this study were confirmed twice, from samples collected at weaning and at sacrifice.

### RT-PCR in Ventral Striatal Samples

**Figure 5:**
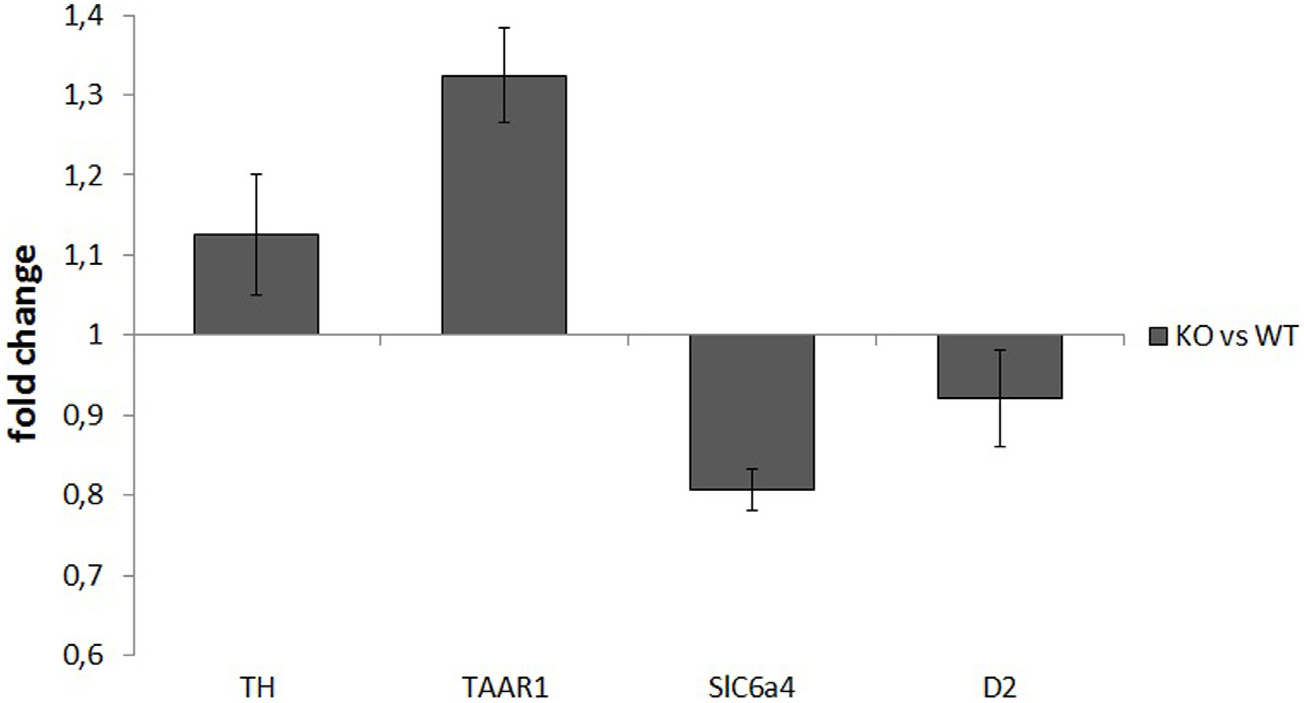
Normalized expression rates [knockout (KO)/wild-type (WT)] of tyrosine hydroxylase (TH), trace amine-associated receptor 1 (TAAR1), serotonin transporter (Slc6a4), and dopamine receptor 2 (D2), in ventral striatum samples of rats. For each of the assayed genes, there were no significant differences in expression levels between WT and KO rats.

mRNA samples used for retrotranscription had a high degree of purity, with an OD260/OD280 ratio of about 2. RT-PCR analysis did not show any significant differences between WT and KO samples in the normalized expression levels (ΔCt) of any of the assayed genes: TH: *P* = 0.52, TAAR1: *P* = 0.51, SERT: *P* = 0.25, D2: *P* = 0.48. ΔΔCt values for the four genes were TH: 1.13 ± 0.08, TAAR1: 1.32 ± 0.06, SERT: 0.81 ± 0.02, and D2: 0.92 ± 0.06 (Figure 5).

## Discussion

Here, we report a preliminary physiological and behavioral profile of DAT KO rats, a new strain generated in IIT (Genova, Italy) and neurochemically characterized by the group of Gainetdinov et al. (1). The weight difference between KO and both WT and DAT–HET rats seems to point to some sort of dwarfism of KO rats. KO rats’ average weight is about 60% of both WT and DAT–HET, but this is clearly not due to differences in food intake, since KO rats eat as much as WT and DAT–HET when kept *ad libitum*. This result is consistent with that already obtained in DAT KO mice (40). It is still not clear if the dwarfism observed in KO rats is caused by an extreme calorie consumption caused by abnormal baseline locomotor activity (1), by a lesser amount of growth hormone (like that observed in KO mice) (40), by some kind of metabolic dysfunction, or by any combination of these factors. Because of this weight anomaly, it was necessary to take many precautions when applying food restriction. If fed with the same amount of food used to keep WT and DAT–HET to 95% of their average body weight, KO rats lost weight more rapidly (data not shown). Therefore, it was necessary to adjust the food quantity administered daily to KO rats, up to a value which ranged between 1.5 and 2 times more than that administered to WT and DAT–HET. Despite eating almost double, KO rats tended to loose most of these calories quite quickly when compared to WT and DAT–HET.

The IDT yielded several unexpected results regarding behavioral abnormalities of DAT KO rats. Even before the beginning of the test, during the 2vs2 training phase, KO rats tended to show a slight preference (i.e., lateralization) for one of the two nose-poke holes, whereas WT and DAT–HET subjects chose at random level during this training phase. Moreover, during the 1vs5 training phase, some KO individuals developed an extreme attraction toward LL hole, reaching sometimes a 100% preference for such a hole. This is uncommon: preference for the LL hole, when there is no delay yet assigned to the large reward, usually hovers around 85–90%, because rats keep a minimum rate of patrolling at the SS hole during sessions (36). More surprisingly, some KO individuals underwent a similar lateralization, but toward SS hole: two of them chose the large reward in less than 10% of total trials and were indeed excluded from the analysis. Rats that were already lateralized toward SS during the training phase were showing little to no interest for LL for the entire duration of the task. Therefore, this abnormal extent of lateralization seems independent from the magnitude of rewards assigned to either of the two holes; this supports the notion that DAT KO rats could be somewhat less sensitive to rewarding stimuli (or less able to redirect behavior toward them). During the 3 weeks of ID testing, KO rats showed a great variability for almost every parameter analyzed, while WT and DAT–HET were instead much more coherent as groups. However, KO as a whole showed some unambiguous features: first, the latency to approach SS hole was always higher than WT and DAT–HET for all sessions throughout the testing phase. Second, at higher delays, the total trials (as well as inadequate nose-poking during the TO) decreased dramatically in both holes. Third, the ones that were strongly lateralized for LL during the training phase maintained an abnormal preference for LL even at higher delays without switching to SS.

These behaviors were completely different from the ones usually observed with rats in this task. WT and DAT–HET, for instance, performed as expected: starting from an LL of about 85% at the end of the 1vs5 training phase, both groups reduced their LL preference and eventually switched toward SS, showing a normal curve of impulsivity. Moreover, WT and DAT–HET rats performed an increasing number of SS trials and of SS inadequate nose-pokes at higher delays. These data provide an insight on reactions clearly due to the aversive effects of the delay: normal subjects experience a sort of “impatience” or “frustration” when forced to wait for the LL reward; thus, they start to perform both more SS inadequate nose-pokes during the TO and more SS trials (i.e., quicker choice after TO has elapsed) when the cost of waiting becomes excessive during LL trials with a very long delay. KO rats, instead, seem to react to the increasing delay with conspicuous consummatory motor stereotypies addressed to the feeding magazine, like sniffing or chewing. A reaction of similar fashion happened in the highly appetitive food test with the paper cups, which were destroyed by KO rats that showed little to no interest for the cheese. As delays assigned to LL became higher, KO rats spent increasingly more time performing these stereotypies overall reducing the number of trials, choosing LL in such few trials, and showing almost no inadequate nose-poking. Oral stereotypies are also presumably behind the increased latency to approach the SS nose-poke hole. When put in the experimental cages, at any stages of the task, KO rats often immediately focused on the feeding magazine and performed their stereotypies, then nose-poking for the first time only after several minutes. To sum it up, DAT KO rats did not show an increased impulsivity when compared to WT and DAT–HET: rather, they displayed a reduced sensitivity to rewarding stimuli, an increased behavioral rigidity, and a number of compulsive behaviors.

When taking the effect of drugs into account, AMPH reduced LL preference and increased number of SS trials in both WT and DAT–HET, as expected: indeed, this psychostimulant drug makes healthy subjects more impulsive and increases motor activity (34, 41). The absence of any noticeable effect of AMPH on DAT KO rats is probably due to the lack of DAT, its main target. On the other hand, RO-5203648 had only slight and nonsignificant effects on KO rats at a 30-s delay, negligibly reducing their elevated LL preference. Considering that the main features of KO rats seem to be a compulsive and rigid attitude, the effect of RO-5203648 would then have been explained with acting on TAAR1 but without the interaction with DAT protein. In line of theory, RO-5203648 could have been able to modulate compulsive symptoms, induced in subjects by a lack of the DAT protein. The reduced preference for the bottle containing a sweet solution, observed in KO rats, could point to a slight anhedonia. This could, in turn, be caused by a reduced sensitivity to the hedonic properties of the sweet water because of an impaired reward system. It is unlikely that these results could have been influenced by a spatial preference for one bottle (though likely in DAT KO rats), because bottles were counterbalanced and swapped daily.

These results surprisingly contradict the findings from a previous study in DAT KO mice (42). As a matter of fact, DAT KO mice showed an increased sucrose preference but the opposite was true for rats. This discrepancy could be due to different sucrose concentrations: it is possible that DAT KO rats are instead more sensitive to the sweet solution, and a 14% sucrose concentration could have acted as an aversive stimulus. However, the hypothesis of a certain form of anhedonia in KO rats could also be supported by the results of the highly appetitive food test. KO rats indeed had eaten less cheese, similarly because of a reduced sensitivity to the rewarding properties of the taste of the cheese. Here, however, another unexpected finding was of extreme interest: KO rats usually ignored the cheese instead of eating it and rather focused on the paper cups, gnawing, eating and/or destroying them. For all the rats tested, there was no latency to approach the cheese once it was presented in the home cage (unformal observations during a task). In other words, even if the cheese was quickly approached, KO rats showed no (or very little) interest toward this fatty and caloric food and started to perform a series of consummatory stereotypies addressed to the paper cups. Only after the paper cups were nearly or completely destroyed, some KO rats started to eat some of the cheese, while others left it utterly untouched.

Thus, KO rats seemed not only less sensitive to rewarding stimuli but also more prone to executing consummatory motor stereotypies. This piece of finding could well point to alterations in the motivational states of KO rats, but also to a different salience assigned to differently meaningful stimuli (i.e., paper cups vs appetitive food). Present male DAT KO rats appear somewhat disturbed by objects they find around, a notion that seems confirmed by an increased marble manipulation in a marble-burying test (33) and by a focused perseverative gnawing of one given metal bar of the top grid, not seen in KO females (unpublished data: informal observation within home cages). It is tempting to speculate that the drive to destroy objects may show some face validity with the profile of boys suffering from CD. When compared to DAT KO mice, who bury less marbles (43), data available at present characterize DAT KO rats as subjects who move marbles around with their mouth (possibly trying to gnaw them) always with a compulsive drive to eliminate them (informal observation by Adinolfi A. and Carbone C.; Master Thesis by Adinolfi A., January 2018). Such behaviors may reflect fundamental aspects for rat models of OCD with comorbid ADHD that are perhaps absent in corresponding DAT KO mice.

Present data can be useful to interpret the many stereotypies that DAT KO rats do have. As far as ADHD is concerned, however, these data point to reward deficiency rather than impairments in attentional processes. Our hypothesis about an increased behavioral rigidity of DAT KO rats seems further supported by results of the Spontaneous Alternation Task (1). Here, KO rats showed a strong lateralization, as they alternated the chosen arms in consecutive trials to less than 50% of times. WT and DAT–HET, instead, alternated as expected to more than 80% of times. The perseverance in choosing the same arm over and over again overlaps the one shown by always choosing the very same nose-poke hole in the IDT, with nearly no consideration for the actual outcome in terms of the correspondent reward.

Many studies (15, 44) showed that the levels of many proteins (like TH, and receptors D1 and D2) are altered in the striatum of DAT KO mice. These studies, however, focused only on proteins, while we wanted to ascertain if similar alterations in DAT KO rats could be due to changes at a transcriptional level. RT-PCR results did not show any relevant difference between WT and KO for all the genes tested (TH, D2, SERT, and TAAR1), suggesting no alterations for these genes, at least at a transcriptional level. Certainly, this finding does not rule out the possibility of a difference in the final protein levels of TH, D2, SERT, or TAAR1. If future studies will find abnormalities in the density of these proteins, like for mice, our present data suggest that they should be due to posttranscriptional alterations. It should be noted that the purity of the initial samples, the satisfying total RNA concentrations, and the concordance of Ct values for each triplicate point to a good quality and yield of the procedures. Thus, the lack of differences between WT and KO should not be attributed to biases. It is possible, however, that, because of the extremely weak expression of TAAR1, a ceiling effect could have masked an actual difference between groups. A replication test with a bigger number of subjects (or with different primers) could yield different results and highlight differences not observed in the present paper (or confirm their absence).

## Conclusion

Even though this study was initially conceived to highlight impulsive behaviors of DAT KO rats, it revealed a compulsive phenotype instead. KO rats are characterized by pervasive consummatory motor stereotypies, noticeable both in their home cages and during tests and operant tasks. Moreover, they seem affected by a strong behavioral rigidity during operant choices. Finally, they show a reduced sensitivity for rewarding properties of food and sweet fluids, although generalization to other natural stimuli like sex or novelty would deserve further work (33). These behavioral abnormalities are not associated with alterations in the expression levels of some genes correlated with DAT (TH, D2, SERT, and TAAR1) in the ventral striatum, although it is possible that the final protein levels could actually differ between WT and KO.

Data obtained with this study provided a first phenotypic characterization of DAT KO rats. First evidences obtained so far [present work; Adinolfi et al. (33); Leo et al. (1)] refer to animals of the various genotypes obtained as offspring of DAT–HET fathers and mothers. DAT KO rats share many features with DAT KO mice: both species present a significant reduction in the total striatum DA levels, also compared to HETs (15, 40); both show an increased locomotor activity and reduced spontaneous alternation in a Y-maze when compared to WTs (15, 45). However, DAT KO rats show a reduced preference to sweet solutions (present data) and manipulate the marbles much more in a burying test, still showing attentional skills in a novelty-preference test (33), while DAT KO mice have an incredibly opposite profile (42, 43). Many other works are warranted to define a better picture of the behavioral and biochemical alterations which affect rats when compared to mice, both lacking the DAT protein. Clarifying the full extent of neurobehavioral alterations in DAT KO rats would be of primary importance to validate them as a model of ADHD, OCD, or other similar neuropsychiatric diseases, like compulsive behavioral addictions. Their use as models in preclinical research is warranted to move steps toward the definition of new and innovative therapies to treat human conditions, especially in adolescents. Indeed, subjects at this age are more vulnerable to malfunctions in the DA circuitry, due to their still incomplete brain maturation (particularly within the prefrontal cortex) and to the developmental challenges they must face: the latter include both the onset of puberty (with its significant physical changes) and the important relational and psychosocial transitions (46, 47).

## Ethics Statement

All experimental procedures were formally approved by the Italian Ministry of Health (formal license 5/2014-B, to GL). Procedures were in close agreement with the European Community Council Directive (2010/63/EEC) and with Italian law. All efforts were made to minimize animal suffering, to reduce the number of animals used, and to utilize alternatives to *in vivo* techniques, if available.

## Author Contributions

SC, FZ, and AP realized the experiments; DL and RG provided the animal model; SC, LC, RT, EA, and GL contributed to writing and commented with their expertise; WA supervised all experiments and writing.

## Conflict of Interest Statement

The authors declare that the research was conducted in the absence of any commercial or financial relationships that could be construed as a potential conflict of interest. The reviewer FP declared a shared affiliation, with no collaboration, with one of the authors DL to the handling editor.
